# The Impact of Preoperative Fasting Duration on Blood Glucose and Hemodynamics in Children

**DOI:** 10.1155/2020/6725152

**Published:** 2020-08-21

**Authors:** Pouran Hajian, Minoo Shabani, Elham Khanlarzadeh, Mahshid Nikooseresht

**Affiliations:** ^1^Department of Anesthesiology, Besat Hospital, Hamadan University of Medical Sciences, Hamadan, Iran; ^2^Student Research Committee, Hamadan University of Medical Sciences, Hamadan, Iran; ^3^Department of Community Medicine, School of Medicine Health Sciences, Hamadan University of Medical Sciences, Hamadan, Iran

## Abstract

**Background:**

Prolonged preoperative fasting is one of the concerns of pediatricians and anesthesiologists in pediatric surgery. The aim of this study was to assess the impact of preoperative fasting duration on blood glucose and hemodynamics in children.

**Methods:**

This cross-sectional study was conducted on 50 children who were between the ages of 3 and 12 years in Besat Hospital, Hamedan, Iran. The time of the last solid and liquid meal taken by child were recorded based on interview with the parents. The first blood glucose test was obtained in the operation room, and the second test was performed 20 minutes after induction of anesthesia by glucometer. Systolic blood pressure (SBP), mean arterial pressure (MAP), and heart rate (HR) were recorded before anesthesia induction and in five-minute intervals in the first 20 minutes of surgery.

**Results:**

The mean age of the children was 6.63 (SD 1.85) years. Mean blood glucose 20 minutes after surgery was 101.17 (SD 92) mg/dl, which was significantly higher than the baseline values (87.66 (SD 11.84) mg/dl) (*P* < 0.001). The comparison of mean blood glucose level between groups of fasting with different duration for solids (<12 hours and >12 hours) and for liquids (<6 hours and >6 hours) revealed no significant difference in either groups (*P* > 0.05). No significant correlation was observed between blood glucose level at the induction of anesthesia with weight and age (*P* > 0.05). There was a significantly negative correlation between duration of fasting for liquids and SBP (*P* > 0.05).

**Conclusion:**

Prolonged preoperative fasting cannot affect blood glucose in children; however, maybe it has impact on systolic blood pressure.

## 1. Introduction

Preoperative fasting is important in reducing the risk of bronchopulmonary aspiration during operation. Mendelson first described preoperative fasting in 1946 from the observation of small population of bronchopulmonary aspiration in pregnant women [1]. In 1987, the Canadian Anesthetists Society introduced the first preoperative fasting recommendations [2].

The guidelines for preoperative fasting have been published by the American Society of Anesthesiologists (ASA) and European Society of Anesthesiologists (ESA). These guidelines are aimed at ensuring acceptable health of pediatric patients and optimizing the experience of surgery in children and their parents [3]. Concerns regarding bronchopulmonary aspiration have limited the administration of free diets before operation. On the other hand, prolonged preoperative fasting is unnecessary in children and may result in thirst, hunger, anxiety, and dizziness, as well as hypoglycemia, headache, agitation, dehydration, electrolyte imbalance, post-operative nausea and vomiting, and increased insulin resistance [4–6]. In normal adults, blood glucose level is maintained during fasting by reduced insulin secretion and increased secretion of glucose regulatory hormones, including growth hormone, glucagon, cortisol, and adrenalin. In contrast to adults, the hyperglycemic reaction in children is less effective, due to lower liver and muscle glycogen storage in children compare to adults, which leads to faster hypoglycemia and ketosis [7, 8].

Serum glucose levels before anesthesia depend on various factors, including age, duration of fasting, and type of premedication. However, the effect of the mentioned factors on the preoperative serum glucose level is still in debate [9]. Age and body weight of children are considered important factors that influence serum glucose levels [10]. Children with lower body weight are more susceptible to hypoglycemia during fasting compared to children with higher body weight [11, 12]. Hypoglycemia reduces brain tolerance to hypoxia and hypotension; thus, hypoglycemic children are prone to complications, including drowsiness, agitation, metabolic acidosis, and seizures. Therefore, hypoglycemia may have negative effects on the growing brain of a child [13].

Prolonged fasting is associated with drop in blood pressure [14] and, therefore, should be avoided in order to prevent hypovolemia and hypotension during anesthesia. The prevalence of hypotension during anesthesia is higher among children who fast for more than 8 hours before operation compared to children with a shorter preoperative fasting period [15].

Prolonged preoperative fasting is one of the concerns of pediatricians and anesthesiologists in pediatric surgery. Although pediatric surgeries are always prioritized in surgery departments, but regarding the number of scheduled surgeries and limitations in the number of operation rooms, preoperative fasting may become longer than the recommended fasting duration by guidelines. The duration of preoperative fasting is not documented in the city of Hamedan before. Due to the importance of the complications of prolonged fasting, this study was conducted to assess the impact of preoperative fasting duration on blood glucose and hemodynamics in children in Hamedan, Iran.

## 2. Methods

### 2.1. Patients

This cross-sectional study was conducted on 50 children who were between the ages of 3 and 12 years and were candidate for elective surgery in the Ear-Nose-Throat (ENT) Department of Besat Hospital, Hamedan, Iran. Sample size was calculated based on the findings of the study by Shah et al. [15] and considering 95% confidence interval for (1-*α*) and 80% power (1-*β*) using the following equation:

(1) $$\begin{array}{c} N=\frac{Z^{2}P\;\left(1-P\right)}{d^{2}}. \end{array}$$

Based on the findings of the previous study, *P* (prevalence of preoperative hypoglycemia) was considered 3.8%, (1 − *P*) = 96.2%, *D* = 0.06, and *Z* = 1.96; therefore, the sample size was calculated as 40.61.

### 2.2. Ethical Considerations

This study was approved by the Ethical Committee of the Hamedan University of Medical Sciences. Parents were notified about the aim of the study and were asked to sign a written informed consent if they agreed to participate in the study. Parents were ensured that no unnecessary laboratory assessment was performed for their children, and the obtained data were recorded anonymously and confidentially.

### 2.3. Inclusion Criteria

Children between the age of 3 and 12 years who were candidate for elective ENT surgery with ASA class 1 and 2 were included in the study.

### 2.4. Exclusion Criteria

Children were excluded if they had cardiopulmonary, hepatic, or any metabolic diseases, diabetes mellitus, endocrine diseases, water and electrolyte disorders, glucose-6-phosphate deficiency, and any type of complications during surgery. Children who had emergency surgery were also excluded.

### 2.5. Data Collection

The time of the last solid and liquid meal taken by child was recorded based on interview with the parents. Body weight was recorded from patient records in a checklist, and the weight for age percentile was identified for each child. No premedication was performed for the children. The first blood glucose test was obtained from each child in the operation room and after primary monitoring and induction of anesthesia. Blood glucose was tested using an express glucometer by using a lancet and a glucometer strip. Second blood glucose testing was performed 20 minutes after induction of anesthesia with a similar method. Systolic blood pressure (SBP), mean arterial pressure (MAP), and heart rate (HR) were recorded before anesthesia induction and in five-minute intervals in the first 20 minutes of surgery using the monitoring machine. Anesthesia induction was performed by administration of 2.5 mg/kg propofol, 0.5 mg/kg atracurium, 1-2 mcg/kg fentanyl, and 0.05 mg/kg midazolam. Anesthesia was maintained by the administration of 1-1.5% isoflurane, oxygen, and 50% nitric oxide. Ringer solution volume was calculated individually for each child. No IV dextrose was administered. Muscle blockade was reversed by administering 0.02 mg/kg atropine and 0.07 mg/kg neostigmine at the end of the surgery. The age of the children, blood pressure, HR, and duration of fasting were recorded in a checklist.

### 2.6. Data Analysis

Descriptive measures including mean and standard deviation (SD) or median were used for continuous variables, including age, blood sugar, SBP, HR, and duration of fasting, based on the Kolmogorov-Smirnov test of normality. Categorical variables were presented using frequency and percentage. Inferential analysis was performed to compare continuous variables, including blood sugar, SBP, MAP, and HR, between fasting duration categories, by using the independent sample *t*-test. The paired *t*-test was performed to compare blood glucose measurements of two measurements. The one-way analysis of variance (ANOVA) was performed to compare mean blood glucose values between weights for age percentile groups. 95% confidence of interval (CI) was used in this study. The Pearson correlation coefficient was calculated to assess the correlation between blood glucose and age, body weight, duration of fasting, blood pressure, and HR and to assess correlation between the duration of fasting and blood pressure and HR. The Spearman correlation coefficient was used to assess the correlation between body weight and blood sugar. Data was analyzed using the statistical package for social sciences (SPSS) software version 16. The level of statistical significance was considered as *P* < 0.05.

## 3. Results

A total of 50 children (58% male and 42% female) participated in the study. The mean age of the children was 6.63 (SD 1.85) years. The mean blood glucose at the induction of anesthesia was 87.66 (SD 11.84) mg/dl. Only one child (2%) had blood glucose less than 50 mg/dl at baseline. Blood glucose values at the induction of anesthesia and 20 minutes afterwards are presented in Table 1. The mean blood glucose 20 minutes after surgery was 101.17 (SD 92 mg/dl), which was significantly higher than the baseline values (87.66, SD 11.84 mg/dl) (*P* < 0.001). The duration of fasting for liquid and solid foods was 9.32 (SD 3.05) hours and 13.44 (SD 3.04) hours, respectively.

In order to compare blood glucose between children with different fasting durations, children were grouped based on the time of the last solid food intake into less than 12 hours and more than 12 hours groups and based on the time of the last liquid intake into less than 6 hours and more than 6 hours groups (Table 2).

Table 2 shows the comparison of blood glucose between groups based on solid/liquid fasting, which revealed no significant difference in either groups (*P* > 0.05) (Table 2). The Pearson correlation coefficient revealed no significant correlation between duration of fasting for solids and liquids with blood glucose at the induction of anesthesia (*P* > 0.05) (Table 3).

The mean body weight of children was 23.05 (SD 8.17) kg. No significant correlation was observed between blood glucose levels at the induction of anesthesia and weight based on age percentiles (*P* > 0.05) (Table 4). In order to compare blood glucose levels between age groups, children were categorized into the 3 to 7.5- and 7.6 to 12-year-old groups. There was no significant difference in blood glucose levels between 3-7.5- (87.26 ± 13.14) and 7.6-12-year-old (88.32 ± 9.63) groups (*P* > 0.05). Overall, no significant correlation was observed between blood glucose levels at the induction of anesthesia with weight and age (*P* > 0.05) (Table 5).

The mean SBP, MAP, and HR at the induction of anesthesia were 104.66 (SD 12.91) mmHg, 76.56 (SD 12.24) mmHg, and 103.14 (SD 11.76) beats per minute, respectively. There was a significant correlation between blood glucose and SBP at the induction of anesthesia (*P* < 0.05) (Table 6). There was a significantly negative correlation between durations of fasting for liquids and SBP. Children in the >6 hour-liquid fasting group had a significantly lower SBP compared to children with <6 hours of liquid fasting. There was no significant correlation between durations of solid fasting and SBP. There was no significant correlation between HR and duration of fasting at the induction of anesthesia (Table 6). There was no significant difference in SBP and HR during surgery (at 5, 10, 15, and 20 minutes) between fasting duration categories for solids (<12 hours vs. >12 hours) and liquids (<6 hours vs. >6 hours).

## 4. Discussion

The aim of this study was to assess the effects of preoperative fasting duration on blood glucose and hemodynamics in children. The current study revealed no significant correlation between body weight and blood sugar. There was also no significant difference in blood glucose between the 3-7.5- and 7.6-12-year age groups. These findings were in line with the findings of the study by O'Flynn [16] and Shah et al. [15].

The current study revealed that the mean duration of fasting for liquids was 9.32 (SD 3.05) hours (4.66-fold more than the standard duration) and for solids was 13.44 (SD 3.04) hours (1.68-fold more than the standard duration). Regarding the type of hospital diet, duration of fasting for solids was compared to 8 hours as the standard duration of fasting. In the study by Arun et al. on 50 children younger than 15 years old, the mean duration of preoperative fasting was 11.25 hours for solids and 9.25 hours for liquids [3]. Similarly, in the study by O'Flynn and Milford [16], the mean duration of preoperative fasting was 14 hours, which was longer than recommendations (in 88% of the children in that study, fasting duration was more than 12 hours and more than 20 hours in 20% of the children) [16]. Similarly, in our study, the duration of preoperative fasting was longer than the ASA guideline recommendation in all of the studied children. Different reports have been recently published regarding the complications of prolonged preoperative fasting. Prolonged fasting disrupts metabolic status of the patient and increases the catabolism and surgical stress [17]. The reasons for prolonged preoperative fasting can be emergency surgery, inconsistency between the parent and hospital in the time of admission, or cancellation of the operation by surgeons.

In the current study, the mean blood glucose was increased 20 minutes after induction of anesthesia. In the study by Shah et al., the mean duration of preoperative fasting was 10.87 (SD 2.68) hours and the mean blood glucose before the induction of anesthesia and 30 minutes after induction of anesthesia was 68.14 (SD 15.23) mg/dl and 110 (SD 34.91) mg/dl, respectively. Hyperglycemia might be due to the effect of surgery stress and increased secretion of glucocorticoids [15]. Some researchers have linked hyperglycemia to peripheral insulin resistance, reduced insulin secretion, or disorder in insulin metabolism. Some other researchers consider hyperglycemia as a defense mechanism to meet glucose requirement of tissues, saving energy and improving intravascular volume through increasing osmolarity. Responsive hyperglycemia in our study was similar to the findings of the previous studies by Das et al. [18], Sharma et al. [19], and Doo et al. [20].

In the current study, blood glucose at the induction of anesthesia was below 50 mg/dl (lower limit of normoglycemia) in one child, who abstained eating solids for 9.45 hours and liquids for 11.95 hours. The preoperative SBP of the child was in a normal range, and the weight for age of the child was above the 25^th^ percentile. The findings of the current study revealed that longer fasting duration was associated with lower blood glucose values before induction of anesthesia which returned to normal range in the next 20 minutes after induction of anesthesia. In a study by Dennhardt et al., the mean fasting duration was 7.8 ± 4.5 hours which differed from the guideline recommendations by 3.3 (SD 2.3) hours. They also reported that the blood glucose was lower in children with longer preoperative fasting duration, but the correlation was not statistically significant [21]. In another study by Kweon et al., the duration of preoperative fasting was reported 10 t0 14 hours in the majority (67%) of the children younger than 7 years old, which was not correlated with preoperative blood glucose [22]. These findings were in line with the findings of our study. In contrast to the findings of our study, Young et al. reported the mean duration of preoperative fasting of 9.86 ± 4.84 hours in their study and reported hypoglycemia in 33 (6%) patients. They also reported that children who experienced episodes of hypoglycemia had longer fasting duration for solid foods [23]. The reason for the difference between the findings of our study and the study by Young et al. might be due to the heterogeneity in sample size, age, or duration of fasting in studies.

Considering the definition of minimum blood pressure in children [24], hypotension was not observed in this study's samples. The findings of the current study revealed that longer fasting duration for liquids was associated with reduced SBP, while the SBP remained within the normal range. Although this correlation was statistically significant, the observed values were not of clinical significance. Furthermore, the mean SBP in children with fasting duration for liquids of more than 6 hours was significantly lower than that in children with a shorter fasting duration for liquids. The reason for this finding is increased dehydration due to reduced fluid intake in children with longer fasting duration for liquids. Pediatric age is considered a susceptible period, and prolonged fasting may have more complications and harms in this age group. It seems that feeding the child with clear liquids does not increase the risk of regurgitation or pulmonary aspiration and can partially improve the psychological state of the child before operation. The ASA and ESA guidelines are followed in most hospitals, but a new approach has recently been proposed for reducing fasting duration. Anderson et al. reported that children who freely consume clear liquids did not experience pulmonary aspiration. In another study, no difference in changes in gastric pH and increase in fluid volume was observed between one-hour fasting on clear liquids and two hours of total fasting.

The current study revealed a significant correlation between blood glucose and blood pressure at the induction of anesthesia and that children with lower blood glucose values at the induction of anesthesia had SBP in the lower limit of normal range although these findings were not of clinical value. One of the limitations of the current study was the inability to perform a case-control study.

## 5. Conclusion

The findings of this study revealed that prolonged preoperative fasting cannot affect hypoglycemia during the operation; however, maybe, it has impact on systolic blood pressure. Regarding the blood pressure, reducing liquid fasting duration and following the current guidelines for fasting duration in pediatric surgeries may prevent hypotension and discomfort in children who are candidates for surgery. Therefore, specialized education of parents and health care providers can be beneficial in preventing prolonged preoperative fasting in children.

## Figures and Tables

**Table 1:** Mean blood glucose values at the induction of anesthesia and 20 minutes after initiation of surgery.

Blood glucose	Mean (SD)	Minimum	Maximum
BS_1_	87.66 (SD 11.84)	49	114
BS_2_	101.92 (SD 17.08)	71	149
*P* value	>0.05		

BS_1_ = blood glucose at the induction of anesthesia; BS_2_ = blood glucose at 20 minutes after initiation of surgery.

**Table 2:** Comparison of blood glucose level in children based on the duration of fasting for solid and liquid foods.

Fasting duration		Mean (SD) of blood glucose	*P* value
Solid fasting	<12 h	87.41 (SD 15.04)	>0.05
	>12 h	87.86 (SD 8.85)	
Liquid fasting	<6 h	91.44 (SD 11.15)	>0.05
	>6 h	86.83 (SD 11.95)	

**Table 3:** Correlation between blood glucose level and fasting duration at the induction of anesthesia.

Correlation with BS_1_	Pearson correlation coefficient	*P* value
Duration of fasting for solids	-0.101	0.487
Duration of fasting for liquids	0.001	0.993

**Table 4:** Comparison of blood glucose levels in children based on weight for age percentiles.

Weight for age percentile	Frequency (%)	Mean (SD) of blood glucose	Minimum blood sugar	Maximum blood sugar
≤5	4 (8)	90.50 (SD 18.95)	69	114
5-25	14 (28)	86.05 (SD 9.44)	68	99
>25	32 (64)	87.81 (SD 12.15)	49	111

**Table 5:** Correlation between blood glucose levels at the induction of anesthesia and body weight and age.

Correlation with BS_1_	Pearson correlation coefficient	*P* value
Body weight	0.016	0.910
Age	0.098	0.499

**Table 6:** Correlation between blood glucose and other hemodynamic parameters.

Correlation	Pearson correlation coefficient	*P* value
Bs_1_/SBP_1_	0.279	0.05
Bs_1_/MAP_1_	0.260	0.01
SBP_1_/liquid	-0.297	0.036
MAP_1_/liquid	-0.183	0.203
SBP_1_/solid	0.145	0.315
MAP_1_/solid	0.122	0.297
HR/liquid	-0.096	0.634
HR/solid	0.142	0.323

SBP_1_ = systolic blood pressure at the induction of anesthesia; MAP1 = mean arterial pressure at the induction of anesthesia; HR1 = heart rate at the induction of anesthesia.

## Data Availability

All data generated or analyzed during this study are included in this published article.
